# Management of potentially inappropriate medication use among older adult’s patients in primary care settings: description of an interventional prospective non-randomized study

**DOI:** 10.1186/s12875-024-02334-3

**Published:** 2024-06-13

**Authors:** Carmela Bou Malham, Sarah El Khatib, Philippe Cestac, Sandrine Andrieu, Laure Rouch, Pascale Salameh

**Affiliations:** 1 Aging Research Team, Center for Epidemiology and Research in POPulation health (CERPOP), Université de Toulouse, Université Paul Sabatier, Inserm, Toulouse, 31000 France; 2 University Paul Sabatier Toulouse III, Toulouse, 31062 France; 3 Department of Pharmacy, Toulouse University Hospitals, Purpan Hospital, Toulouse, 31059 France; 4 School of Medicine, Lebanese American University, Byblos, 1401 Lebanon; 5 University of Nicosia Medical School, Nicosia, 1065 Cyprus; 6 Faculty of Pharmacy, Lebanese University, Hadath, 1100 Lebanon; 7 Institut National de Santé Publique, Epidémiologie Clinique et Toxicologie INSPECT-LB), Beirut, 1100 Lebanon

**Keywords:** Pharmacists, Inappropriate prescribing, Ambulatory care, Aged

## Abstract

**Background:**

The management of inappropriate medication use in older patients suffering from multimorbidity and polymedication is a major healthcare challenge. In a primary care setting, a medication review is an effective tool through which a pharmacist can collaborate with a practitioner to detect inappropriate drug use.

**Aim:**

This project described the implementation of a systematic process for the management of potentially inappropriate medication use among Lebanese older adults. Its aim was to involve pharmacists in geriatric care and to suggest treatment optimization through the analysis of prescriptions using explicit and implicit criteria.

**Method:**

This study evaluated the medications of patients over 65 years taking a minimum of five chronic medications a day in different regions of Lebanon. Descriptive statistics for all the included variables using mean and standard deviation (Mean (SD)) for continuous variables and frequency and percentage (n, (%)) for multinomial variables were then performed.

**Results:**

A total of 850 patients (50.7% women, 28.6% frail, 75.7 (8.01) mean age (SD)) were included in this study. The mean number of drugs per prescription was 7.10 (2.45). Roughly 88% of patients (*n* = 748) had at least one potentially inappropriate drug prescription: 66.4% and 64.4% of the patients had at least 1 drug with an unfavorable benefit-to-risk ratio according to Beers and EU(7)-PIM respectively. Nearly 50.4% of patients took at least one medication with no indication. The pharmacists recommended discontinuing medication for 76.5% of the cases of drug related problems. 26.6% of the overall proposed interventions were implemented.

**Discussion:**

The rate of potentially inappropriate drug prescribing (PIDP) (88%) was higher than the rates previously reported in Europe, US, and Canada. It was also higher than studies conducted in Lebanon where it varied from 22.4 to 80% depending on the explicit criteria used, the settings, and the medical conditions of the patients. We used both implicit and explicit criteria with five different lists to improve the detection of all types of inappropriate medication use since Lebanon obtains drugs from many different sources. Another potential source for variation is the lack of a standardized process for the assessment of outpatient medication use in the elderly.

**Conclusion:**

The prevalence PIDP detected in the sample was higher than the percentages reported in previous literature. Systematic review of prescriptions has the capacity to identify and resolve pharmaceutical care issues thus improving geriatric care.

**Supplementary Information:**

The online version contains supplementary material available at 10.1186/s12875-024-02334-3.

## Introduction

Prescribing quality is an important determining factor for the well-being of elderly populations [1, 2]. Inappropriate medication use can lead to an increased risk of adverse drug events (ADE) ranging from minor to life-threatening [2–5]. Polypharmacy, the chronic use of 5 or more medications, is a common practice among elderly patients and is a well-known risk factor for poor drug compliance, medication‐induced harm, and hospital admission [6–8]. Potentially inappropriate medication use can be mitigated by evaluating the prescription process [9–11].

Ensuring appropriate medication use remains a challenge for primary healthcare professionals. To fight this iatrogenic problem, studies propose various ways to assess and reduce potentially inappropriate drug prescribing (PIDP) [12]. Proposed clinical strategies can be categorized as explicit (criteria-based) tools and implicit (judgment-based) tools. Explicit criteria are lists of indicators for potentially inappropriate drugs and/or diseases, agreed upon by consensus of experts, to avoid in the geriatric population. Interventions using explicit criteria can effectively reduce PIDP [13], but they often neglect overall patient characteristics. On the other hand, implicit tools are based on clinical judgment and combine research data with clinical evaluation [14]. The drug prescription for each subject is analyzed individually, with a personalized assessment of the benefit/risk ratio of each drug with regard to co-morbidities and other prescribed drugs.

Comprehensive geriatric assessment is the complete, global assessment of the older adult to develop an individualized medication therapy plan in collaboration with the patient and the healthcare team [15, 16]. In this multidisciplinary process, pharmacists, specifically, play a crucial role in performing a medication review for older adults and using an approach that combines explicit and implicit criteria. The combination of the implicit and explicit methods mediated by this patient-centered approach demonstrates a reduction in the risk of ADE in older adults and ensures a better reflection of the patient’s overall drug management [2, 17, 18].

The French Society of Clinical Pharmacy developed a systematic process by establishing a medication review form that can be implemented by either the community or hospital pharmacist in primary care settings in order to evaluate patient’s prescriptions and detect inappropriate medication use through applying both implicit and explicit criteria. At the end of the process, pharmacists were able to suggest pharmaceutical interventions and communicate them to the treating physcian [17]. This project entitled MGPIDP-L (Management of potentially inappropriate drug prescribing among Lebanese patients in primary care settings) introduced the same systematic process to reduce inappropriate prescriptions and medication use for older people with multimorbidity and polypharmacy in Lebanon. The pharmacist used the adapted tools of the project as a support to facilitate and standardize the analysis process.

Lebanon is a country on the eastern coast of the Mediterranean. It currently has the highest percentage of older adults aged 65 years and above (7.4%) among Arab countries [19, 20]. This proportion has been increasing since 1990 (at 5.2%) and is expected to reach 21% in 2050 [21, 22]. To our knowledge, there is no robust study evaluating the benefits of a complete assessment tool that contributes to optimizing prescriptions for the elderly in primary care settings in Lebanon. In addition to that, existing studies were retrospective and were limited in their analysis to the use of explicit criteria: Beers list alone [23–26] or in combination with Screening Tool of Older Person’s Prescriptions (STOPP)/Screening Tool to Alert to Right Treatment (START) [27]. One study relied on the implicit criteria supplemented with only the Beers list as explicit criteria [28] .

### Aim

This project described the implementation of a systematic process for the management of potentially inappropriate medication use among Lebanese older adults. Its aim was to involve pharmacists in geriatric care and to suggest treatment optimization. The primary objective of our study was to evaluate the medication use among older adults in Lebanese primary care settings using the combination of the implicit approach and the explicit approach with the latest versions of Beers criteria [29–31], Laroche list [32], European Union 7 (EU(7)-PIM list) [33], STOPP START criteria [34], and STOPP Frail list [35]. Our secondary objectives were to assess disease-related factors, the patient’s behavioral management of his treatment, drug/therapy-related factors, the proposed pharmaceutical interventions, and the satisfaction of the patients after performing the medication review.

### Ethical approval

The study was approved by the Institutional Review Board of the National Institute of Public Health, Clinical Epidemiology, and Toxicology-Lebanon under the number of 2021REC-001-INSPECT-09-04. It was conducted according to the principles expressed in the Declaration of Helsinki and followed the international standards pertaining to protection of human research subjects. Patients were required to demonstrate an understanding of the study protocol and to sign an informed consent form. A form was obtained from all of the patients.

## Methods

### Design

The MGPIDP-L project was a prospective, multicentered, interventional study conducted in different regions of Lebanon over a period of 12 months, spanning from [January 2020] to [January 2021]. The project targeted different primary care settings: upon hospital discharge, at the patient’s home, and in community pharmacies. All community and hospital pharmacies in Lebanon were invited to participate and be co-investigators in the study. Contact with the hospital/community pharmacists was established at conferences or through phone calls or mail to explain the rationale, objectives, and methodology of the study. Hospital pharmacists who showed interest were asked to select recently discharged patients who met the inclusion criteria and to be part of the medication review process. On the other hand, community pharmacists who agreed to participate were requested to promote the project by displaying a poster in their pharmacies, thus encouraging patients to participate.

The medication review was conducted by experienced clinical pharmacists CBM and SK after obtaining the patient’s consent.

### Sample size and population selection

The sample size was calculated using Epi Info 7.2.5.0. Based on recent statistics, the population of Lebanese older adults is estimated to be 765,000. As a result, for a confidence level of 99.9% and a prevalence of 60%,1038 patients were needed [27].

Inclusion criteria encompassed older adult patients aged 65 or above and taking five or more chronic medications per day regardless of their overall health status and degree of dependency. Eligible patients should be able to provide all necessary medical information (comorbidities, medications such as dose and route of administration, frailty, behavioral management towards their medical treatment, side effects, self- medication) directly or with the assistance of a caregiver or family member in certain cases such as cognitive deficiency. Additionally, patients were required to demonstrate an understanding of the study protocol and to sign an informed consent form.

### Procedure

Patient interviews were conducted in private medical settings, and their names were undisclosed to ensure confidentiality. To determine potentially inappropriate medication use, two experienced clinical pharmacists CBM and SK conducted a drug utilization review for each patient. These pharmacists underwent specific training as well as specific courses to be able to perform medication reviews for older adults. A third consulting pharmacist PS resolved disagreements between the two primary evaluators. The strategy involved: a patient interview, an analysis of the appropriateness of drug use in terms of indication, dosage, safety, and efficacy, a medication synthesis, and a formulation of justified pharmaceutical interventions intended for the patient’s general practitioner. Effective communication either by mail or phone calls between physician and pharmacist was essential during this process to make rational decisions about medications and patient needs. After the physician’s approval, every intervention was communicated to the patient.

Drugs were coded according to the Anatomical Therapeutic Chemical classification system [36], therapeutic classes, and pharmacological classes [37] on the medication review form as well as on SPSS.

### Outcome measures

Inappropriate medication use can involve a problem related to the prescribed drugs (PIDP) and/or drugs used without a medical prescription and/or an undertreated medical condition and/or an undertreated vaccination.

A PIDP was defined as a prescription that contains at least one drug problem identified by one of the used criteria and can be due to the inappropriate prescribing of the physician or the inappropriate use of the prescribed drug by the patient. Medication use was analyzed by combining explicit and implicit criteria.

The implicit criteria were based on the global assessment of the patient and took into account whether the prescription corresponds to an indication or need (Drug utilization review) [38].

As part of the explicit criteria, five different lists for potentially inappropriate medications in the geriatric population were used: Beers criteria [29–31], Laroche [32], STOPP START criteria [34], STOPP Frail list [35], and EU(7)-PIM [33].

The Beers criteria, initially developed by an expert consensus panel in 1991 in the United States (US), are the most widely cited criteria used to assess inappropriate drug prescribing. However, they are applicable only to medications available in the US market and do not discuss drug-nutrient interactions, medication underuse, over-the-counter medications, or medication adherence. Likewise, the tool lacks clear recommendations for appropriate dosing and dosing frequency [29, 30]. The STOPP criteria were introduced in 2008 and updated in 2015. STOPP primarily helps in the detection of drug-drug interactions and duplication of drugs within a class. The Irish list STOPP START specifically addresses medication underuse [34]. STOPP Frail list applies only to patients ≥ 65 years old and having: end-stage disease, a life expectancy of < 1 year, a severe impairment of physiological and/or cognitive functions, or in the palliative treatment [35].

The French list Laroche, developed in 2007, was the first list to propose safer therapeutic alternatives and took into account the medication redundancy [32]. The EU(7)-PIM list, a screening tool developed collaboratively by experts from seven European countries, allows the identification and comparison of potentially inappropriate medication patterns for older people across European countries. The latest versions of these lists were used in our study [33]. Additionally, recommendations of good clinical practice provided specifically for the elderly by the French High Authority of Health (HAS), including the Clinical Practice Indicators and Alert and Mastering of Iatrogenesis, were also included in the analysis [39].

### Data collection and questionnaire

During the patient interview, the pharmacist used a medication review form that was originally developed by the SFPC. The original French medication review form was translated forward and backward in Arabic and English respectively, to maintain the equivalence of the survey in the target language. The translations were piloted to address any mistranslation or ambiguity. This newly adapted medication review form provided a framework for the collection of information about current medication use, patient history, frailty, drug management, and patient beliefs about their medication and compliance with prescription instructions [7, 8, 40, 41]. Specific information about the medical treatment of every patient was also collected such as the drug name, dose, route, and frequency of administration were recorded on paper.

The medication review was composed of: (1)A sociodemographic component to assess the lifestyle of the patient (2)A medical component listing all co-morbidities, medical/surgical history (3)Find’s auto-questionnaire that will assess the notion of frailty or robustness of the subject (involuntary weight loss of more than 4.5 kg, fatigue felt by the patient, speed walk, sedentary lifestyle, decrease in muscle strength. (4)Part concerning the medications, this table lists all the medications prescribed, their dosage, their frequency, their indication with regard to previous antecedents and co-morbidities (5)A part allowing the pharmacist to evaluate the patient’s ability to manage his/her medications (6)One aspect on adherence, adverse effects and self-medication(7)A section to evaluate patient’s satisfaction about the medication review process, using a scale from 0 = dissatisfied to 10 = very satisfied and 9 questions concerning the levels of satisfaction “Figure S1”.

### Statistical analysis

SPSS version 21 was used to perform the statistical analysis. Before starting the descriptive analysis, data weighting was done in order to take into consideration the distribution of the geriatric population in Lebanon according to sex and region based on the Lebanese central database of statistics. This helped in adjusting our sample’s composition to be reflective of the population’s composition [42, 43].

Descriptive statistics for all the included variables were performed using mean and standard deviation (Mean (SD)) for continuous variables and the number of observations and percentage (n, (%)) for multinomial variables.

The reliability of the newly created satisfaction scale was conducted. In addition to that, the validity test was calculated to ensure the consistency and accuracy of the survey.

## Results

### Included sample

A sample of 850 patients were enrolled during the 12-month inclusion period from [January 2020] till [January 2021]. 188 (18.1%) Records for patients with incomplete, insufficient, or conflicting data were excluded. Ten community pharmacies from different regions of Lebanon and four hospitals were included in this study.

### Reliability

The Cronbach’s alpha for the items of the patient’s satisfaction was 0.864. This indicates a high level of internal consistency among the items measuring patient satisfaction, which strengthens the reliability of the satisfaction scale.

### Validity

Concurrent validity testing was performed to compare the English, Arabic, and French versions of the survey by the three investigators, and the answers were totally matching and consistent. The same patient answered the three versions with the same response thus ensuring the validity of our survey.

### Descriptive results of the population

#### Sociodemographic characteristics

Among the 850 patients, 431 were women (50.7%). The older adult population can be divided into three life-stage subgroups: the young-old (approximately 65–74), the middle-old (ages 75–84), and the old-old (over age 85) [44], young-old patients accounted for almost half of the sample size (48%) while 34.9% were middle-old, and 17.1% were old-old.

The distribution in the different provinces was 38% in Mount Lebanon, 18.9% in North, 16.8% in Beirut, 14.5% in the South, and 11.8% in Bekaa. Weighting was used to adjust the latter variable to represent the population from which the sample was selected [43]. The medication review was conducted on 51.3% of patients upon discharge from the hospital, and the rest were conducted in outpatient settings “Table 1”.

**Table 1:** Sociodemographic characteristics (*N* = 850)

Variables		*n* (%)
Gender	Men	419 (49.3)
	Women	431 (50.7)
Age (Mean (SD))		75.73 (8.0)
Age class	65–74	408 (48.0)
	75–84	297 (34.9)
	≥ 85	145 (17.0)
Weight in Kg (Mean (SD))		76.22 (12.7)
Residence Area (Mouhafaza)	Beirut	143 (16.8)
	Mount Lebanon	323 (38.0)
	South	123 (14.5)
	Bekaa	100 (11.8)
	North	161 (18.9)
Type of Housing	Alone	100 (11.8)
	With spouse	213 (25.1)
	With family	537 (63.2)
Assistance	House keeper	177 (20.8)
	Registered Nurse	32 (3.8)
	Physiotherapist	32 (3.8)
	Senior meal delivery	13 (1.5)
	Other aid	17 (2.0)
Context	Upon hospital discharge	436 (51.3)
	Per treating physician order	117 (13.8)
	Lack of compliance	11 (1.3)
	Poly medication	221 (3.0)
	Other (Patient’s request)	65 (7.7)

#### Clinical characteristics of patients

Frailty was calculated using Auto-find questionnaire that takes into consideration several factors. 28.6% of patients were frail, and 30.5% of the sample were dependent. The highest percentage of the alerting signs and symptoms in geriatrics was attributed to daytime drowsiness (49.8%) and insomnia (42.1%) “Table S1”.

The number of comorbidities ranged from 1 to 10 with a mean of 3.51 (1.6) per prescription. Diseases of the cardiological system as well as nutritional and metabolic disorders were the most reported ones among the participants. In particular, the highest reported comorbidities were hypertension (79.5%), dyslipidemia (48.8%), diabetes (41.7%), coronary insufficiency (28.1%), and osteoporosis (19.2%) “Table S2”.

#### Characteristics of medications used

The total number of medications used by the population ranged between 5 and 23 with a mean of 7.10 (2.45) and a median of 6 medications per patient. 14.2% of patients were hyperpolymedicated using 10 or more concurrent different drugs [45].

90.6% of the medications were used chronically versus only 1.7% for acute conditions. On the other hand, 7.6% were classified as auto medication that is used by the patient without a prescription.The leading class of medications used was the cardiovascular agents ((*n* = 2130) 35.93%) followed by vitamins, minerals, and electrolytes (*n* = 633)(10.68%)) “Table S3”.

Half of the subjects (50.2%) took their medications on their own without any assistance. 20.7% of the patients were willing to take a medication without a prescription and 7.1% modified the dose without consulting the physician. The majority of the patients took their medications on a regular base whereas, 17.1% of them frequently skipped a dose “Table S4”.

### Potentially inappropriate medication use

Inappropriate medication use can concern a problem related to the prescribed drugs (PIDP) and/or drugs used without a medical prescription and/or an undertreated medical condition and/or undertreated vaccination.

Among the 850 included patients, 95.7% (*n* = 813) had at least one issue related to potentially inappropriate medication use.

#### Prevalence of undertreated medical conditions

Undertreated problems were related either to an undertreated indication/condition as demonstrated in “Table S5” or an undertreated vaccination based on international guidelines [46, 47].

64.7% of the patients had at least one undertreated medical condition. As seen in “Table S5”, undertreated diseases were either related to the absence of therapy for a valid medical condition (71.4%) or the absence of a complementary medication as per guidelines (28.6%). The most common undertreated condition was osteoporosis. A low percentage of the geriatric population received therapy for the prevention of osteoporosis. It was followed by myocardial infarction being 13.7% of the cases whereby the complete treatment was not being prescribed; an antiplatelet, beta blocker, angiotensin converting enzyme inhibitor (ACEI), or a statin when needed [48].

As for the vaccination, we evaluated the participants who were not vaccinated with influenza and tetanus boosters and found 87.1% versus 94.1% respectively. On the other hand, 8.3% of the eligible participants received their shot of pneumococcal vaccine according to international guidelines.

#### Prevalence of potentially inappropriate drug prescribing

“Table 2” presents the number of medical prescriptions that contain at least one type of drug related problem or in other terms PIDP identified by one of the used criteria. 88% (*n* = 748) of the participants suffered from at least one PIDP.

74.9% of the problems can be classified as misuse (according to expert opinions, contraindication, drug-drug interactions, side effects, pharmacodependance, drug monitoring, not first line), 23.2% as overuse (overdosage, redundancy, no indication HAS indicators), and 1.9% as underuse [49] “Table 2”.

**Table 2:** Number of medical prescriptions containing at least one type of problem (*N* = 850)

Type of problem	Potentially inappropriate drug prescriptions	*n* (%)
Prescription containing at least one medication listed in^a^	Beers criteria	564 (66.4)
	Laroche	233 (27.4)
	EU (7)-PIM	547 (64.4)
	STOPP START criteria	488 (57.4)
	STOPP Frail list	6 (0.7)
	Other expert opinion	19 (2.2)
	Contraindication	2 (0.2)
	Underdosage	55 (6.5)
	Overdosage	36 (4.2)
	No indication	428 (50.4)
Concomitant prescription HAS indicators ^a^	≥ 3 psychotropic medications	2 (0.2)
	≥ 2 antipsychotics	1 (0.1)
	≥ 2 Benzodiazepines	1 (0.1)
	≥ 2 antidepressants	4 (0.5)
	≥ 2 diuretics	21 (2.5)
	≥ 4 antihypertensives	50 (5.9)
	Redundancy of the same drug	123 (14.5)
	Drug/drug interaction	88 (10.4)
	Drug not taken	0 (0.0)
	Side Effects	58 (6.8)
	Pharmacodependance	1 (0.1)
	Drug Monitoring	2 (0.2)
	Not first-line treatment ^b^	142 (16.7)

^a^ Explicit criteria

^b^ The initial, preferred, or best treatment for a disease. It is often the therapy that combines the best efficacy with the best safety profile and/or the lowest cost [50]

Several tools were used to detect these problems: explicit criteria (67.4%) and implicit criteria (32.6%).

66.4% (*n* = 564) and 64.4% (*n* = 547) of the patients had at least 1 drug with an unfavorable benefit-to-risk ratio according to one of the following lists of PIDPs: Beers criteria and EU(7)-PIM respectively.

9.3% of the subjects had a prescription that did not comply with the recommendations of the French Health Authority guidelines (HAS).

Nearly 50.4% had at least one medication with no indication, 14.5% of had redundancy in the prescription, and 10.4% experienced at least 1 significant drug-drug interaction.

“Table 3” represents the inappropriate drugs frequently prescribed for the most common components of PIDP. Classes of medications most involved in PIDP were anti-inflammatory agents, proton pump inhibitors (PPIs), and cardiovascular medications like the angiotensin receptor inhibitor (ARB) and ACEI.

**Table 3:** Classes of medications identified to cause the most frequent PIDP (*N* = 850)

Components of PIDP	Drug Class	Non-Proprietary Name	ATC	*n* (%)
Drug with unfavorable benefit to risk ratio according to three lists (Beers criteria + EU(7)-PIM + STOPP START criteria)	Anti-inflammatory agents	Ibuprofen	M01AE01	20 (2.4)
		Diclofenac	M01AB05	27 (3.2)
	Gastrointestinal agents	Omeprazole	A02BC01	131 (15.4)
		Esomeprazole	A02BC05	63 (7.4)
		Rabeprazole	A02BC04	39 (4.6)
		Lansoprazole	A02BC03	18 (2.1)
No indication	Blood	Acetylsalicylic acid	B01AC06	110 (12.9)
	Products/Modifiers/Volume expanders	Clopidogrel	B01AC04	13 (1.5)
	Gastrointestinal agents	Omeprazole	A02BC01	124 (14.6)
		Esomeprazole	A02BC05	48 (5.7)
		Rabeprazole	A02BC04	30 (3.5)
		Lansoprazole	A02BC03	17 (2.0)
≥ 4 prescribed antihypertensives per prescription	Cardiovascular agents	Moxonidine	C02AC05	12 (1.4)
		Valsartan	C09CA03	12 (1.4)
		Amlodipine	C08CA01	32 (3.8)
		Hydrochlorothiazide	A10BA02	38 (4.5)
		Bisoprolol	C07AB07	36 (4.2)
		Candesartan	C09CA06	16 (1.9)
Redundancy of the same drug	Blood	Acetylsalicylic acid	B01AC06	50 (5.9)
	Products/Modifiers/ Volume expanders	Clopidogrel	B01AC04	59 (6.9)

### Proposed interventions

Various types of pharmaceutical interventions were proposed for each type of potentially inappropriate prescription whether related to a drug, undertreated vaccination, or to an undertreated condition “Table 4”.

**Table 4:** Proposed interventions related to potentially inappropriate medication use (*N* = 850)

	Type of Intervention	*n* (%)
Undertreatment interventions	Add a Complementary/Corrector Drug	801 (94.2)
Drug-related interventions	Dosage adjustment	89 (10.5)
	Route of administration	0 (0)
	Improve dispensing/and or administration	1 (0)
	Therapeutic follow up	304 (35.8)
	Add a new prescription	1 (0)
	Change a drug	306 (36)
	Stop or refuse to deliver	650 (76.5)
Vaccine-related interventions	Recommending a Pneumococcal vaccine	487 (57.3)
	Recommending a Flu vaccine	740 (87.01)
	Recommending a Tetanos vaccine	800 (94.1)

Suggested interventions involve incorporating the necessary complementary medication into the prescription. As discussed before, the two highest undertreated comorbidities were osteoporosis and myocardial infarction. Vitamin D was recommended as a complementary drug for osteoporosis prevention, while the treatment for myocardial infarction that were added were ACEI, beta blockers, or statins when needed [48].

On the other hand, the most common drug-related intervention was to stop the dispensing of a specific medication that is being used inappropriately (76.5%). Someof the medications that were recommended to be stopped were non-steroidal anti-inflammatory agents and PPIs as they were the most frequently overused classes. 26.6% of these interventions were accepted while taking into consideration that we didn’t obtain feedbacks concerning proposed vaccine interventions.

The majority of the accepted interventions were related to a discontinuation of a drug due to a redundancy in the prescription. Drowsiness and hypotension were common side effects felt by our subjects due to the presence of 4 antihypertensive agents in one prescription. In most of the cases an antihypertensive agent was discontinued, thereby improving these symptoms.Physicians also accepted to add vitamin D for the patient for osteoporosis and fall prevention, while they hesitated to discontinue non-steroidal anti-inflammatory agents and proton pump inhibitors.

### Satisfaction of patients

The satisfaction score ranged from 0 to 10 with 10 being really satisfied. The mean score was 7.27(1.4).

The satisfaction depended on the patient’s opinion concerning the duration of the interview and its benefits in terms of understanding of drugs, management, and preparation of medications “Table S6”.

Patients were highly satisfied because they were able to manage their medications using the counseling tips provided by the pharmacists. Even psychologically, they were feeling much better because we took the time to listen to all their complaints.

## Discussion

The aim of our study was to manage potentially inappropriate prescribing among the Lebanese geriatric population in primary care settings. To achieve this, we used both implicit criteria and explicit criteria, incorporating the latest versions of Beers criteria [29–31], Laroche list [32], EU(7)-PIM list [33], STOPP START criteria [34], and STOPP Frail list [35]. The rate of PIDPs (88%) detected using both approaches, was much higher than the rates reported in different countries such as France, United States of America, and Canada [14, 51, 52]. The prevalence of PIDP was higher than studies conducted in Lebanon where it varied from 22.4 to 80% depending on the explicit criteria used as mentioned earlier, the settings, and the medical conditions of the patients. Some studies report this percentage in inpatient settings [25, 26]. Others focus on percentages in certain medical conditions such as heart failure [23] and advanced chronic kidney diseases [26]. On the other hand, we integrated heterogeneous criteria from the five different lists to improve the detection of all types of PIDP since Lebanon obtains drugs from many different sources and countries. Simultaneous use of both criteria improved the detection of PIDP, decreased the risk of ADEs, and enhanced the appropriate use of medications. In addition to that, this high percentage can be explained by the fact that there’s a limited number of geriatric specialists and the lack of a standardized tool for the assessment of outpatient medication use in the elderly [19].

The present study confirmed the high prevalence of hypertension, dyslipidemia, and diabetes among the older Lebanese population. These are the most common comorbidities in Lebanese primary care settings even when compared to studies in other countries [53]. The mean number of drugs per prescription (7.10(2.45)) is typical for such patients [23, 54]. Notably, 10.7% of participants took an excessive 10 or more medications that are highly associated with inappropriate prescribing [13, 14, 16, 18]. This highlights the contemporary relevance of addressing and mitigating polypharmacy issues in healthcare practices.

The most frequent PIDP was the prescription of at least 1 drug with an unfavorable benefit/risk ratio according to one of the lists of potentially inappropriate medications. Anti-inflammatory drugs were the most common drug category with an unfavorable benefit/risk ratio. Diclofenac and acetylsalicylic acid were the most associated with unfavorable ratios due to their high risk of gastrointestinal bleeding, ulceration, or perforation, which may be fatal [55]. In addition to that, we detected the presence of dual antiplatelet treatment in many prescriptions, especially in myocardial infarction. It was identified as a redundancy in the prescription because the treatment was continued beyond the approved duration [48].

The second category of drugs involved in PIDP with an unfavorable ratio and no specific indication were related to the digestive tract and metabolism (i.e., PPIs), as observed in previous studies [23, 25, 27, 56]. The use of PPIs has important benefits in the treatment of upper gastrointestinal tract disorders and in conditions of excess acid secretion such as the use of certain drug classes, a specific infection, and others. They are widely used particularly in the elderly especially among those taking nonsteroidal anti-inflammatory drugs and corticosteroids [57]. Older adults are often prescribed PPIs for unclear indications and for long periods. PPIs were also prescribed to overdose even when we found a valid indication (prevention of ulcers or prevention of gastrointestinal bleeding in patients on antithrombotic treatments) or it was used for a long duration [27, 58, 59]. Different studies have highlighted an increased rate of adverse events (decreased vitamin and mineral absorption, osteoporotic-related fractures, pneumonia, Clostridium difficile infection) with long-term PPIs use and overdosing [56–59].

Drug interactions involved PPIs (i.e., esomeprazole, lansoprazole) with an antiplatelet agent (i.e., clopidogrel). A pharmacological interaction between clopidogrel and some PPIs has been proposed based on mutual CyP450-dependent metabolism, but the influence on clinical outcomes has been conflicting [60]. Despite this inconsistency, a clinically important interaction cannot be definitively excluded, particularly among patients with higher overall cardiovascular risk [48, 60]; therefore, it has been considered a PIDP in this study.

The use of more than four antihypertensives, an alert from HAS, was also common. The combination of ARBs, Betablocker, calcium channel blocker, and alpha 2/ imidazoline receptor agonist [39].

Undertreated conditions were another major issue especially incomplete therapy for myocardial infarction (absence of ACEI, Beta blockers, or statin when needed [48]. The use of statins for the prevention of coronary artery disease,particularly of myocardial infarction (MI), is based on some well designed strategies aimed at treating both asymptomatic high-risk patients (primary prevention) or patients with established coronary artery disease (secondary prevention) [48]. Osteoporosis was also highly undertreated [61]. The main problem was that even though guidelines recommend preventing osteoporosis in the elderly population [47], preventive treatment is absent or incomplete. Hypercholesterolemia was also undertreated since many Lebanese people prefer getting a lifestyle modification instead of taking medication.

As individuals get older, their immune systems weaken over time. This is why vaccination is considered to be an essential topic to discuss especially that it is rarely evaluated in Lebanon. According to the guidelines, older adults are recommended to get the seasonal flu (influenza), tetanus, diphtheria, pertussis, shingles, and pneumococcal vaccines. On the other hand, guidelines recommend having the pneumococcal vaccine if the patient has one of the following conditions: heart failure, chronic respiratory failure, obstructive pulmonary disease, emphysema, severe asthma under prolonged corticosteroid therapy, chronic liver diseases (alcoholic or not), cirrhosis, end-stage renal failure, diabetes not balanced by lifestyle modification, and a history of invasive pneumococcal disease [46, 47]. . The study highlights a concerning low rate of vaccination among participants. Our results emphasized the considerable gap between observed vaccination practices and the established guidelines.

While analyzing the prescription, we took into consideration if the patient’s medical treatment was adapted to his lifestyle as well as his/her behavior towards drug management. Every patient was asked about his behavioral management towards his/her medical treatment such as his/her compliance, preparation, storage, self-medication, and others. These factors were missing in published literature. It is essential to evaluate them because they help us ensure that the medications are taken properly and achieve their planned, therapeutic outcomes.

We went further in the study to propose interventions to the physician in order to improve the treatment. The rate of accepted interventions was low due to many reasons such as misconceptions regarding the roles and responsibilities of other team members, physician’s insufficient time, and lack of financial compensation. The health sector in Lebanon is facing a lot of challenges due to the instability in the country. Hospitals are struggling to recruit and retain qualified healthcare workers who are choosing to leave the country. In addition to that, healthcare professionals are also experiencing many obstacles to do their jobs like in the case of medication shortages. Despite the presence of the above reasons that blocked our physician-pharmacist collaboration, our study was the first interventional in Lebanon to suggest modifications to the patient’s prescription as compared to other articles on this subject.

### Strengths

Our study has several strengths. Data were collected by the same healthcare professionals throughout the study. They underwent adequate professional development. We piloted an intervention for the management of prescriptions of elderly patients using comprehensive criteria. We combined explicit and implicit criteria to optimize pharmaceutical analysis, as well as the five different lists. We demonstrated the efficacy of this method in capitalizing on the experience of the patient’s treating physicians and pharmacists and the latest literature. We also encouraged the multidisciplinary approach to exchange information and optimize the prescription of the elderly thus spreading awareness, and we integrated pharmacists in the process of reviewing geriatric medical prescriptions.

Our intervention had the desired effect of detecting inappropriate prescriptions and reducing patients’ overall number of prescriptions. In addition, it had the effect of increasing patient satisfaction with their overall treatment.

### Weaknesses

Nevertheless, our study has some limitations. Eventhough we tried to gather all the information; some data were missing from the medical record wich may lead to information bias. The information that were also gathered in community settings were sometimes incomplete due to the absence of digital patient profile. Heterogeneous criteria were used to define our principal outcome, so it may be difficult to compare our results with those of other studies because our method may overestimate the PIDP rate.In the MGPIDP-L study, older adults were included on a voluntary basis. Therefore, it may have resulted in a selection bias, favoring the participation of people more interested and more aware of medical research. In addition to that, the results of our study cannot be generalized to the aged patients across Lebanon, because this study only took into consideration outpatients in primary care settings. We were faced with a difficulty in establishing communication with the physicians. The low rate of acceptance of interventions may be due to the politicial and economic crisis that Lebanon is facing as well as insufficient time due to the busy work schedule.These problems and challenges will be taken into account for future studies.

## Conclusion

The management of medications in geriatric populations stands to benefit from wraparound care from pharmacists and physicians. The risk of adverse effects, especially in Lebanon, increases across all health settings. Clear definitions of metrics for evaluations can illuminate the extent of inappropriate medication use and encourage communication among healthcare workers. This study demonstrates the potential application of our measurement tool in identifying and evaluating PIDPs. The goal of this manuscript was to demonstrate potential applications in community pharmacist workflows.

Future directions exploring the specific applications of this evaluation tool would benefit from narrowing the scope of research. Rather than genericizing the tool to work in outpatient and community care settings, the tool can be streamlined for community pharmacists. One potential benefit from this application is empowering community pharmacists to initiate communication with physicians on behalf of their patients. This application stands to be quite beneficial for lower resourced communities where the informal relationships between community caretakers and physicians provide necessary health education to patients.

### Electronic supplementary material

Below is the link to the electronic supplementary material.

Supplementary Material 1

## Data Availability

All relevant data are within the paper, its supporting information file, and on the repository Zenodo at the following DOI 10.5281/zenodo.6321171.

## List of abbreviations

SD: Standard deviation
US: United States
PIDPs: Potentially inappropriate drug prescribing
ADE: Adverse Drug Events
HAS: Haute Autorité de Santé
SFPC: Société Française de Pharmacie Clinique
MGPIDP-L: Management of potentially inappropriate drug prescribing among Lebanese patients in primary care settings
STOPP: Screening Tool of Older Person’s Prescriptions
START: Screening Tool to Alert to Right Treatment
PPI: Proton Pump Inhibitor
ARB: Angiotensin Receptor Inhibitor
ACEI: Angiotensin Converting Enzyme Inhibitor
ATC: Anatomical Therapeutic Chemical
