# Supplementary material for: Management of potentially inappropriate medication use among older adult’s patients in primary care settings: description of an interventional prospective non-randomized study
Source: BMC Prim Care. 2024 Jun 13;25:213. doi: 10.1186/s12875-024-02334-3 (PMC11170768; doi:10.1186/s12875-024-02334-3)
Supplement: Supplementary file 1 — Supplementary Material 1 [file 12875_2024_2334_MOESM1_ESM.docx]

**Supplementary Materials**

**Supplementary Materials**

The following figures and tables are presented in this section: the English version of the used medication review tool (Figure S1), clinical characteristics of the patient (Table S1: geriatric syndromes including falls, frailty, urinary incontinence, loss of appetite, weight loss, insomnia), types of comorbidities of the participants (Table S2 : neoplasms, cardiovascular diseases, gastrointestinal problems, inflammatory and immune system disorders, metabolic and endocrine system related conditions, mental health, neurological comorbidities, renal and urogenital diseases, comorbidities related to the respiratory system, and other conditions), different classes of medications that are used by the participants (Table S3: analgesics, antibacterials, antidepressants, antigout, anti-inflammatory agents, blood glucose regulators, blood products, cardiovascular agents, gastrointestinal agents, genitourinary agents, respiratory agents, nutrients, electrolytes, and others), behavioral management of the medical therapy (Table S4: general management of the medical treatment, self medication, preparation and administration of medications, side effects), undertreated conditions (Table S5), and the satisfaction of the patients with regard to the medication review (Table S6: duration of the survey, benefits of the conducted survey, additional questions).

Figure S1: The English version of the medication review tool


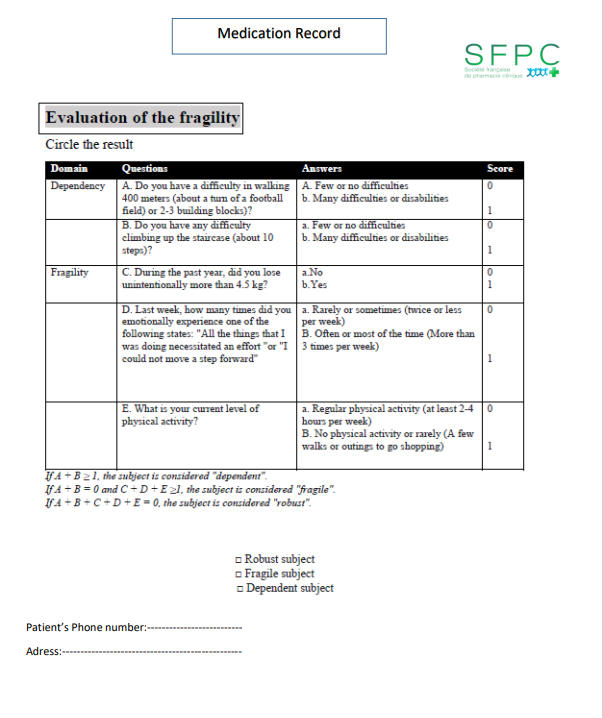


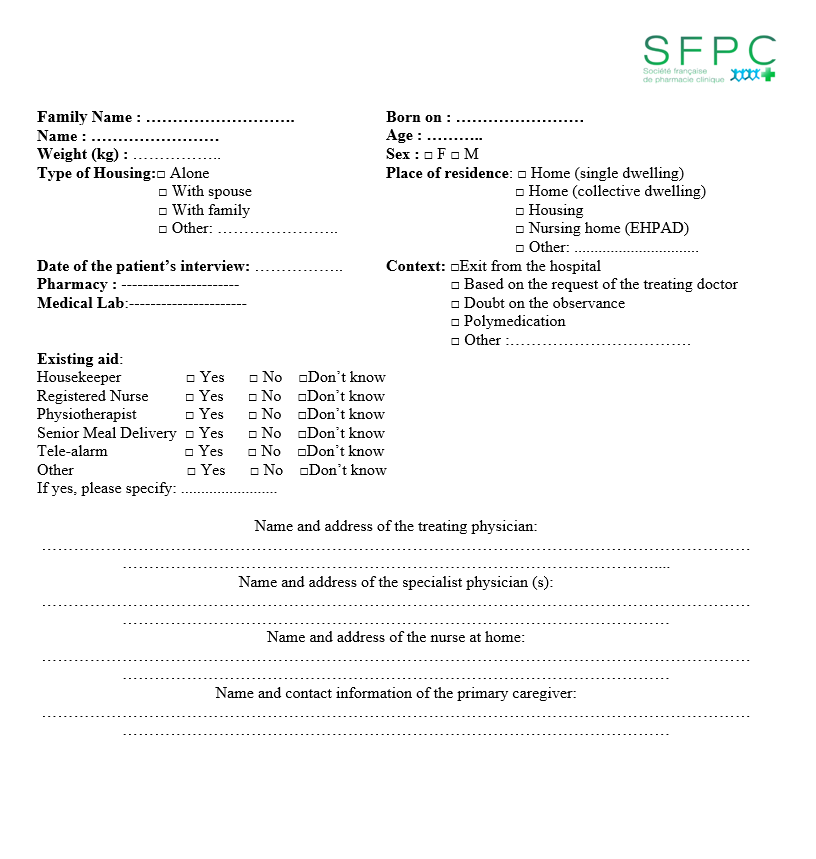


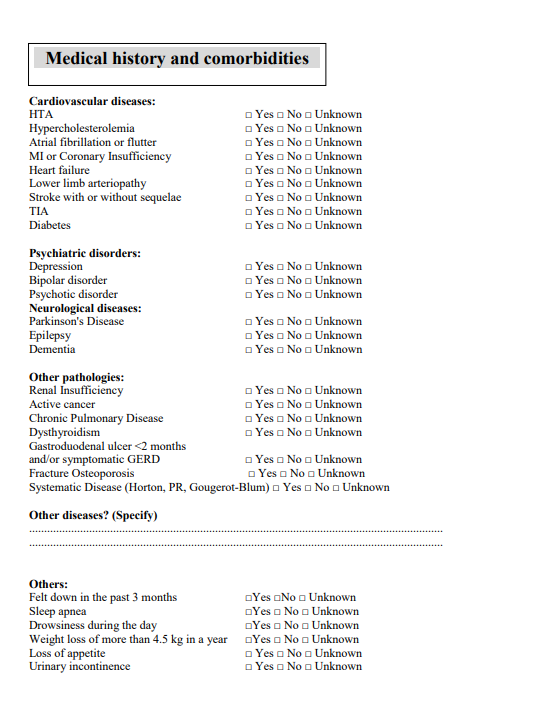


**
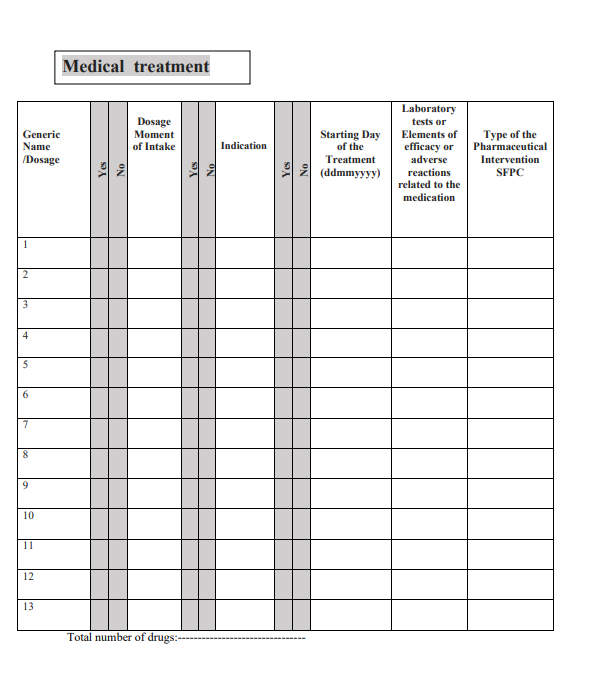
**

**
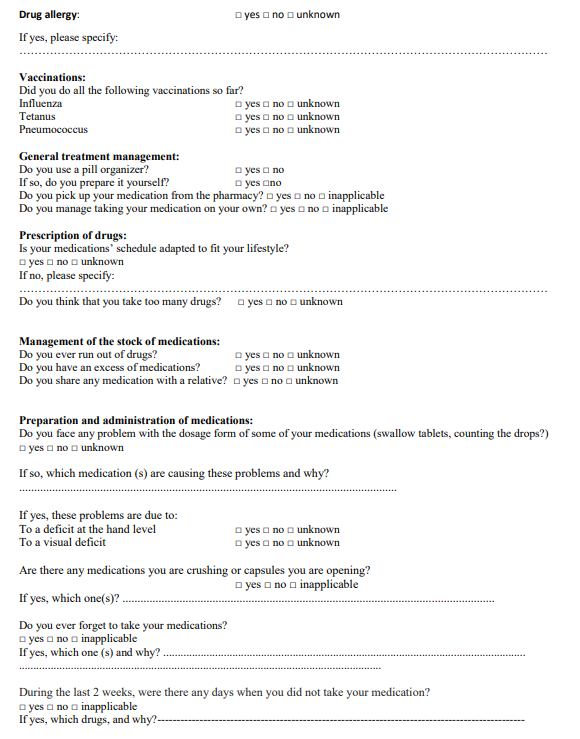
**

**
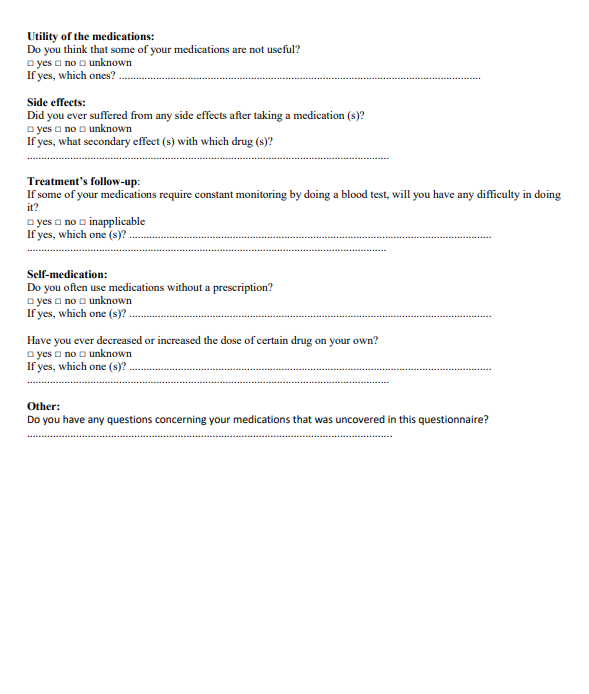
**

**
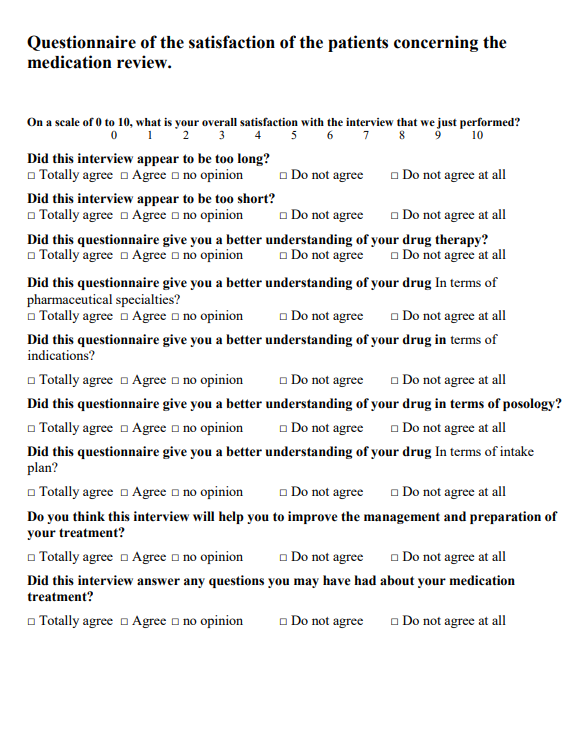
**

Table S1: Clinical characteristics of patients (N=850)

| **Clinical Characteristics** | | **n (%)** |
| --- | --- | --- |
| **Fragility Status** | **Robust** | 348 (40.94) |
|  | **Dependent** | 259 (30.47) |
|  | **Fragile** | 243 (28.59) |
| **Felt down in the past 3 months** | | 166 (19.53) |
| **Insomnia** | | 358 (42.12) |
| **Daytime drowsiness** | | 423 (49.76) |
| **Weight loss > 4.5 kg/year** | | 205 (24.12) |
| **Loss of appetite** | | 206 (24.24) |
| **Urinary incontinence** | | 200 (23.53) |
| **Drug Allergy** | | 63 (7.41) |

Table S2: Comorbidities of the participants (N=850)

| **Comorbidities** | | **n (%)** |
| --- | --- | --- |
| **Cancer and neoplasms** | Active cancer | 16 (1.88) |
| **Cardiovascular** | Atrial Fibrillation | 131(15.41) |
|  | Heart Failure | 110 (12.94) |
|  | Hypertension | 676 (79.53) |
|  | Lower Limb Arteriopathy | 12 (1.41) |
|  | Angina or Myocardial Infarction | 240 (28.24) |
|  | Stroke | 41 (4.82) |
|  | Transient Ischemic Attack | 16 (1.88) |
| **Gastrointestinal** | GERD^a^ or ulcer**<** 2 months go | 72 (8.47) |
|  | Hepatic Failure | 0 (0.00) |
| **Inflammatory and immune system** | Systemic Disease (Horton, Crohn’s disease, , Gougerot-Blum, Rheumatoid arthritis) | 9 (1.06) |
| **Metabolic and endocrine** | Diabetes | 354 (41.65) |
|  | Dyslipidemia | 415 (48.82) |
|  | Dysthyroidism | 60 (7.06) |
|  | Hyperuricemia | 66 (7.76) |
| **Mental Health** | Bipolar Disorder | 4 (0.47) |
|  | Depression | 93 (10.94) |
|  | Psychotic disorder | 50 (5.88) |
| **Musculoskeletal** | Osteoporosis | 163 (19.18) |
| **Neurological** | Alzheimer/dementia | 21 (2.47) |
|  | Epilepsy | 19 (2.24) |
|  | Parkinson | 16 (1.88) |
| **Renal and urogenital** | Renal Failure | 55 (6.47) |
| **Respiratory** | Pulmonary disease^b^ | 99 (11.65) |
| **Other Comorbidities or conditions** | Benign Prostate Hypertrophy | 68 (8) |
|  | Pain | 52 (6.12) |
|  | Vertigo | 50 (5.88) |
|  | Other | 127 (14.94) |

^a^ Gastroesophageal reflux disease, ^b^ Pulmonary disease including asthma, chronic obstructive pulmonary disease

Table S3: Therapeutic classes of the used medications (N=5929)

| **Therapeutic Classes** | **n (%)** |
| --- | --- |
| **Analgesics** | 235 (3.96) |
| **Anti bacterials** | 60 (1.01) |
| **Antidepressants** | 115 (1.94) |
| **Antigout** | 76 (1.28) |
| **Anti-inflammatory** | 68 (1.15) |
| **Blood Glucose Regulators** | 606 (10.22) |
| **Blood Products/Modifiers/Volume Expanders** | 681 (11.49) |
| **Cardiovascular Agents** | 2130 (35.93) |
| **Gastrointestinal Agents** | 421 (7.10) |
| **Genitourinary agents** | 191 (3.22) |
| **Respiratory Tract Agents** | 259 (4.37) |
| **Therapeutic Nutrients/Minerals/ Electrolytes** | 633 (10.68) |
| **Other** | 309 (5.21) |

Table S4: Drug management behavior of the participants (N=850)

|  | **Drug Management Behavior** | **n (%)** |
| --- | --- | --- |
| **General treatment management** | Do you use a pill organizer? | 113 (13.29) |
|  | If so, do you prepare it yourself? | 70 (8.24) |
|  | Do you pick up your medication from the pharmacy? | 350 (41.12) |
|  | Do you manage taking your medication on your own? | 427 (50.24) |
| **Prescription of drugs** | Is your medications’ schedule adapted to fit your lifestyle? | 720 (84.71) |
|  | Do you think that you take too many drugs? | 307 (36.12) |
| **Management of the stock of medications** | Do you ever run out of drugs? | 96 (11.29) |
|  | Do you have an excess of medications? | 100 (11.76) |
|  | Do you share any medication with a relative? | 54 (6.35) |
| **Preparation and administration of medications** | Do you face any problem with the dosage form of some of your medications (swallow tablets, counting the drops?) | 68 (8) |
|  | If yes, these problems are due to a deficit at the hand level | 14 (1.65) |
|  | If yes, these problems are due to a deficit at the visual level | 8 (0.94) |
|  | If yes, these problems are due to a deficit at both level | 10 (1.18) |
|  | Are there any medications you are crushing or capsules you are opening? | 37 (4.35) |
|  | Do you ever forget to take your medications? | 145 (17.06) |
|  | During the last 2 weeks, were there any days when you did not take your medication? | 75 (8.82) |
| **Utility of the medications** | Do you think that some of your medications are not useful? | 56 (6.59) |
| **Side effects** | Did you ever suffered from any side effects after taking a medication (s)? | 85 (10) |
| **Treatment’s follow-up** | If some of your medications require constant monitoring by doing a blood test, will you have any difficulty in doing it? | 64 (7.53) |
| **Automedication** | Do you often use medications without a prescription? | 176 (20.71) |
|  | Have you ever decreased or increased the dose of certain drug on your own? | 60 (7.06) |

Table S5: Undertreated comorbidities (N=850)

|  | **Comorbidities** | **Add a Synergistic/ Corrector Drug (n)** | **Absence of Therapy for a Valid Medical Condition (n)** | **Total (%)** |
| --- | --- | --- | --- | --- |
| **Cardiovascular** | Hypertension | 5 | 8 | 1.53 |
|  | Atrial Fibrillation | 29 | 8 | 4.35 |
|  | Myocardial Infarction | 90 | 26 | 13.65 |
|  | Heart Failure | 34 | 8 | 4.94 |
|  | Stroke | 11 | 4 | 1.76 |
|  | Transient Ischemic Attack | 2 | - | 0.24 |
| **Gastrointestinal** | GERD | - | 4 | 0.47 |
| **Metabolic and endocrine** | Diabetes | 1 | 7 | 0.94 |
|  | Dyslipidemia | 3 | 66 | 8.12 |
|  | Dysthyroidism | - | 2 | 0.24 |
|  | Hyperuricemia | - | 7 | 0.82 |
|  | Mental Health | - | - | - |
|  | Depression | - | 17 | 2 |
|  | Psychotic Disorder | - | 1 | 0.12 |
| **Musculoskeletal** | Osteoporosis | 40 | 401 | 51.88 |
| **Renal and Urogenital** | Renal Failure | 2 | 2 | 0.47 |
| **Respiratory** | Pulmonary Disease | 8 | 4 | 1.41 |
| **Others** | (Anemia, Benign Prostate Hypertrophy…) | 3 | 5 | 0.94 |

TableS6: Patient’s satisfaction of the medication review tool

|  | **Do not agree at all** | **%** | **Do not agree** | **%** | **No opinion** | **%** | **Agree** | **%** | **Totally agree** | **%** | **Mean (SD)** |
| --- | --- | --- | --- | --- | --- | --- | --- | --- | --- | --- | --- |
| Did this interview appear to be too long? | 44 | 5.18 | 96 | 11.29 | 331 | 38.94 | 157 | 18.47 | 222 | 26.12 | 3.49(1.14) |
| Did this interview appear to be too short | 41 | 4.82 | 128 | 15.06 | 402 | 47.29 | 254 | 29.88 | 25 | 2.94 | 3.11(0.87) |
| Did this questionnaire give you a better understanding of your drug therapy? | 2 | 0.24 | 9 | 1.06 | 302 | 35.53 | 399 | 46.94 | 138 | 16.24 | 3.78(0.73) |
| Did this questionnaire give you a better understanding of your drug In terms of pharmaceutical specialties? | 1 | 0.12 | 10 | 1.18 | 301 | 35.41 | 425 | 50 | 113 | 13.29 | 3.75(0.70) |
| Did this questionnaire give you a better understanding of your drug in terms of indications? | 1 | 0.12 | 14 | 1.65 | 300 | 35.29 | 417 | 49.06 | 119 | 14 | 3.75(0.71) |
| Did this questionnaire give you a better understanding of your drug in terms of posology? | 1 | 0.12 | 15 | 1.76 | 289 | 34 | 426 | 50.12 | 118 | 13.88 | 3.76(0.71) |
| Did this questionnaire give you a better understanding of your drug In terms of intake plan? | 1 | 0.12 | 14 | 1.65 | 301 | 35.41 | 427 | 50.24 | 107 | 12.59 | 3.73(0.70) |
| Do you think this interview will help you to improve the management and preparation of your treatment? | 1 | 0.12 | 13 | 1.53 | 305 | 35.88 | 417 | 49.06 | 115 | 13.53 | 3.74(0.71) |
| Did this interview answer any questions you may have had about your medication treatment? | 2 | 0.24 | 15 | 1.76 | 311 | 36.58 | 403 | 47.41 | 119 | 14 | 3.73(0.72) |
| On a scale from 0 to 10 how much were you satisfied from the interview? | | | | | | | | | | | 7.27(1.39) |
